# Agricultural Insurance and Agricultural Economic Growth: The Case of Zhejiang Province in China

**DOI:** 10.3390/ijerph192013062

**Published:** 2022-10-11

**Authors:** Shaolong Zeng, Bingying Qi, Minglin Wang

**Affiliations:** School of Economics, Hangzhou Normal University, Hangzhou 311121, China

**Keywords:** agricultural insurance, agricultural economic growth, agricultural insurance premium revenue, influence, China

## Abstract

Based on the theories of welfare economics, this paper analyzed the mechanism of agricultural insurance (AI) affecting agricultural economic growth (AEG), theoretically, and carried out an empirical analysis by using the random effects model and thirteen years of panel data, which included the annual data of 11 cities in Zhejiang Province, China, from 2007 to 2019. The gross output value of agriculture, forestry, animal husbandry, and fishery (GOVA) of 11 cities in Zhejiang Province is selected as the explained variable, agricultural insurance premium income (AIPI) as an explanatory variable. We selected area of waterlogging removal (AWR), rural electricity consumption (REC), total power of agricultural machinery (TPAM), and crop-sown area (CSA) as control variables. The study shows that: (1) the AIPI has a significant positive impact on the growth of GOVA. When other conditions remain unchanged, a 1% increase in AIPI increases the GOVA by 0.166%, accordingly; (2) The control variables of REC, TPAM, and CSA are statistically significant for the growth of the GOVA. The elasticity coefficient of REC is 0.325, the elastic coefficient of the TPAM is 0.287, and the elasticity coefficient of CSA is −0.281.

## 1. Introduction

### 1.1. Research Background

Agricultural insurance (AI) provides protection for economic losses caused by natural disasters, accidents, epidemics, diseases, etc., in the process of agricultural production (AP). It can promote the growth of the agricultural economy (AE), effectively disperse the risks of agricultural disasters to farmers, expand the scale of agricultural industry development, and have a significant positive impact on the upgrading of the agricultural industry structure. According to the guidance on the development of AI in China’s “No. 1 central document” for 2022, the government has clarified the importance of AI in the process of rural vitalization, and proposed some important measures as below: the coverage of AI should be further expanded, AI and reinsurance should be actively developed, the “insurance + futures” model should be improved and upgraded, the market-based sharing and compensation of agricultural credit risks should be strengthened, and the role of agricultural credit guarantees should be improved. In 2021, China’s agricultural insurance premium income (AIPI) reached 97.6 billion CNY, a year-on-year increase of nearly 19.8%, providing risk protection for 180 million households of more than 4.7 trillion CNY.

Zhejiang Province, China started a policy-based agricultural insurance (PAI) pilot program in 2006, which is the earliest reform pilot. The PAI has created a model based on “co-insurance management” in accordance with the principle of “government promotion + market operation + farmers’ voluntariness”. In March 2015, the “Regulations on Agri-cultural Insurance” of Zhejiang Province was formally implemented. Zhejiang’s AIPI has continued to grow from 862 million CNY in 2016 to 1.262 billion CNY in 2019. In 2020, the AIPI of Zhejiang Province (excluding Ningbo) was 1.111 billion CNY, 118 AI types were carried out, 49.3 billion CNY were provided for risk protection, there were 805.8 thousand insured households, payment of 788 million CNY was made in insurance claims, 112.8 thousand households were benefited, and the simple compensation rate was 71%.

On 26 August 2021, Zhejiang Province issued the “Implementation Opinions on Accelerating the High-quality Development of Agricultural Insurance”, which proposes the goals that, by the end of 2022, the PAI coverage rate of various staple crops in Zhejiang reached more than 70%. In addition, the PAI basically covered the main varieties of planting, aquaculture, and forestry in the whole Province. The coverage rate of pig breeding insurance reached more than 90%, the AI depth, which means the percentage of premium in the added value of the primary industry, reached 1%, and the agricultural insurance density, which means the value of premium divided by the agricultural population, reached 500 CNY/person. The insurance guarantee basically covers basic insurance liabilities, such as natural disasters, and gradually expands from guaranteeing production risks to guaranteeing market risks.

### 1.2. Research Problem and Significance

There is much literature that take analyses the importance and effect of AI. AI is a significant driver of agricultural development worldwide [1], as it is one of the management risk tools [2,3] that is now widely recognized as an important means of protection for production and for the life of people in rural areas [4]. AI has a special social and ecological significance, wherein it is the driver of rural development at the same time [5], and it can play an impactful role in reducing uncertainty and, consequently, increasing investment [6]. Due to the complexity of agricultural business, risks in agriculture are as important to society as to the individual farmers [7]. It is of great importance for farmers to have AI in order to ensure economic sustainability [8], as it is considered a promising instrument to manage climate risks and to enhance the food security of smallholder farmers [9]. Crop insurance might be a vital tool to mitigate agricultural risk [10], as it has a significant positive impact on agricultural green total factor productivity (AGTFP) [11]. Managing the risks of climate variability on crop production is central to ensuring financially viable farming systems and sustainable food production [12]. Meanwhile, AI can increase the relative importance of biological pesticides [13]. The Insurance Plus Futures policy pilot in agricultural price reform is a leading indicator of reform in China’s AP and rural finance architecture [14].

With a global market of 30 billion USD, AI plays a key role in risk finance and contributes to climate change adaptation by achieving Sustainable Development Goals, including no poverty, zero hunger, and climate action [15]. Each country is unique in terms of its risk characteristics [16]. China’s current agricultural policy includes a wide range of agricultural subsidies, but the overall effect and levels of protection are low [17]. The effect of policy-oriented AI on increasing farmers’ income has always been controversial [18]. Farmers systematically undervalue agricultural index insurance [19]. Agricultural producers face a variety of significant risks; historically, only government-subsidized products have achieved widespread adoption [20].

With the rapid development of China’s AI, there are some shortages, such as unbalanced regional development and a lack of characteristic types of AI. AI plays a more important role in eastern China and non-major crop-producing areas [1]. The AI subsidy in China’s central and western regions with a relatively low level of economic development is dominated by government financial premium subsidies, while China’s eastern regions with a relatively high level of economic development should seek diversified forms of subsidies other than premium subsidies.

In 2006, Zhejiang Province launched and gradually expanded the pilot program of policy-oriented AI, which is the first province in China to implement AI. Meanwhile, Zhejiang is located on the east coast of China, and its agriculture is greatly affected by natural disasters such as typhoons, so the role of AI is more important. There is little literature on AI in Zhejiang Province in China. Therefore, it is important to study the relation between agricultural insurance and agricultural economic growth by taking the case of Zhejiang Province in China.

The theoretical significance of this paper is as follows: based on theories of welfare economics, it logically analyzes the mechanism of AI affecting AEG and provides theoretical supplements for boosting the AE and assisting the rural revitalization strategy. The practical significance of this paper lies in the fact that it plays a vital role for Zhejiang Province to continuously strengthen services and innovations in AI, improve the coverage and security level of AI, and contribute to the wisdom and strength of AI on the progress of rural “common prosperity” construction.

### 1.3. Research Content and Innovation

This paper provides an empirical analysis on the influence of AI on agricultural output (AO) and agricultural economic growth (AEG), by using the latest data on AI and AEG, selects AIPI as an explanatory variable, and area of waterlogging removal (AWR), rural electricity consumption (REC), total power of agricultural machinery (TPAM), and crop-sown area (CSA) as control variables. This paper discusses the future development path of AI in Zhejiang Province, divided into three different subjects—the government, insurance companies, and farmers—and from various perspectives, including optimizing the financial subsidy structure, improving the AI system, developing new AI products, strengthening AI publicity, and improving farmers’ confidence in the insurance company.

The contents of this paper are structured as follows. Section 2 provides a literature review and Section 3 analyzes the theoretical basis and mechanism of AI affecting AEG. Section 4 presents the status quo of AI and AEG in Zhejiang Province. Section 5 gives an empirical analysis of AI’s influence on AEG, including variable selection, hypothesis, empirical result, and lagging effect test. Section 6 provides conclusions and policy recommendations.

This paper focuses on the influence of AI on AO and AEG. With the rapid development of China’s AI, AI plays a more important role in eastern China and non-major crop-producing areas. There is little literature on AI in Zhejiang Province in China. Based on theories of welfare economics, this paper contains a theoretical analysis of the mechanism of AI affecting AEG, providing theoretical supplements for boosting the AE and assisting the rural revitalization strategy. It plays a vital role for Zhejiang Province to continuously strengthen services and innovations of AI, improve the coverage and security level of AI, and contribute to the wisdom and strength of AI on the progress of rural “common prosperity” construction.

## 2. Literature Review

This paper elucidates the relevant research on AI and its impact on agricultural economic development in recent years.

### 2.1. Impact of Agricultural Insurance on the Investment, Technology Adoption, and Production of Agriculture

AI can positively promote AP and economic growth among agricultural households [21]. AI led to a significant increase in aggregate AO across China’s provinces [1]. The AI subsidy policy encourages farmers to expand their production scale by mitigating production risks [22]. Crop insurance promotes agricultural economic growth if the insurance mechanism is introduced into the risk model; premium subsidies constantly improve AO [23]. The development of AI, induced by premium subsidies, significantly promotes primary industry production, and primarily affects agriculture and husbandry among the four subindustries of the primary industry [24]. These AI subsidies have a significant positive effect on food security [17]. Upscaling weather index insurance (WII) programs may help to spur agricultural development in the small farm sector [25]. Environmental protection and AI are complementary mechanisms of risk protection that provide significant support to agricultural entrepreneurship and the development of AP [26].

AI can promote farmers’ investment, technology adoption, and AGTFP too. Insurance can play an impactful role in reducing uncertainty and, consequently, increasing investment [6]. Insurance helps to diminish the risk-averse farmers’ suboptimal input due to the presence of uncertainty [27]. Weather index insurance has a significant effect on the technology adoption of rural households [28]. The increase in AI can improve AGTFP [29]. AI has a U-shaped effect of on AGTFP in the context of a large country through three channels: environmentally friendly production, labor inputs, and operation scale [30].

### 2.2. Classification of Agricultural Insurance and Regional Differences in Its Impact

AI can be classified into different kinds based on different insurance objects. Individual crop insurance includes potato insurance [31] and cold-weather damage PAI contracts for tea trees (an economic crop) in the Zhejiang Province of China [32]. Insurance pilots based on weather indices [33], excessive rainfall index insurance for sugar cane farmers [12], weather index insurance [34], and agricultural climate index-based insurance (IBI) compensate farmers for losses from adverse climatic conditions [35]. Index insurance is often promoted as a solution to many of the barriers that are thought to limit the supply of formal insurance coverage to smallholder farmers and livestock owners in developing countries [36]. Income insurance generates significant risk reduction at a lower cost than other programs such as index insurance or warrantage [37]. Most AI products are heavily subsidized by governments [38].

AI has a different impact on different regions and farmers. The policy effect is heterogeneous in different regions [22]; it plays a more important role in eastern China and nonmajor crop-producing areas [1]. The total cost insurance pilot program increases farmers’ income, compared with central and western regions, and farmers’ income is more likely to increase in the eastern regions [39]. The effect of crop insurance in promoting AGTFP is stronger for cash crops compared with food crops [11]. Crop-producing farms with an AI contract are more efficient than farms without this risk management tool [40].

### 2.3. Impact of Agricultural Insurance on the Agricultural Environment

AI may bring environmental change. On the one hand, it has a positive influence. Purchasing AI can encourage farmers to increase the use of biological pesticides and reduce the use of chemical pesticides [13]. The implementation of policy-supported AI significantly reduced the amount of agricultural fertilizers used and nonpoint source pollution in China [41]. On the other hand, some scholars hold opposing views. The AI subsidy policy has negatively impacted the agricultural environment [22]. At the national level, AI contributes to agricultural carbon emissions, thereby weakening agricultural carbon welfare performance [42]. The pilot of PAI has aggravated the nonpoint source pollution of agricultural fertilizers in China [43]. The increase in AI can aggravate air pollution to a certain extent [29].

### 2.4. Impact of Agricultural Insurance on Poverty Reduction

There is a threshold for poverty alleviation resulting from AI, as AI does not help peasants escape deep poverty, because premiums keep them below the threshold; meanwhile, premium subsidies strengthen the poverty reduction resulting from insurance, as the increased income and the elimination of risk move some peasants above the critical threshold [44]. Policy-oriented AI is beneficial to the increase in farmers’ income on the whole, but it has significant heterogeneity on farmers of different income groups, and its influence becomes greater with the increase in farmers’ income [18].

### 2.5. Factors Affecting Farmers’ Willingness to Take Out Agricultural Insurance

The factors affecting farmers’ willingness to take out AI were farmers age, education, land size, sources from which they receive information on AI, the amount of support paid by the state, and the amount of debt, pure product, and agricultural income [2]. Among the reasons why farmers take out AI are insuring their crop, avoiding any potential loss, securing their income, and presence of disaster risk. The main reasons why farmers do not take out AI is the registration and share problems of their lands [45]. The tendency to conform with peers and learning by imitation have become new influencing factors that affect farmers’ purchases of policy-based planting AI [46]. The factors affecting farmers’ uptake of insurance are the amount of hazelnut production, non-agricultural income status, farmer’s agricultural experience, total agricultural land assets, and the profitability of hazelnut production [8]. Farmers’ risk aversion significantly increases the probability of their decision to buy weather index-based crop insurance [47]. The current insurance system impasse demonstrates that the producer does not accept policies that do not convert into an immediate income benefit [48]. Individual riskiness is positively related to willingness to pay for agricultural index insurance [19]. Delayed payments lowered farmers’ trust in insurance as a risk management option [49]. AI affordability and risk perception and management are the major influencing factors [50]. Compared with farm income, non-farm income is associated with an increased (decreased) demand for insurance among households in eastern (western) China [21]. In northeast Thailand, 65% of farmers’ income relied on the non-agricultural sector, which might be the one reason for constraints of insurance sales [51].

### 2.6. Difference of Farmers’ Preference for Insurance

Farmers have different preferences for AI. Farmers prefer AI that covers a wide range of disasters, including output price drops and input cost risks; farmers who have suffered plant disease and insect and pest damage are willing to pay a high premium for the insurance with a high compensation ratio and several types of crops covered; full-time farmers and large-scale farmers prefer insurance products with a low-complexity claims procedure [31]. Farmers with less experience and a high income tend to choose “60% of full-cost insurance product”. Farmers with a lack of specialization and lower diversified planting tend to choose “full-cost insurance product”. In contrast, farmers with higher education prefer “output value insurance product” [52]. The farmers’ decision to use AI and cooperatives was positively correlated, farmers who purchased AI mainly used to mitigate production risks were more likely to join agricultural cooperatives, which are more used to sharing market risks, and vice versa [53]. Education level, access to technical assistance, use of management tools, and farm size positively affect the probability of adopting AI. In addition, farmers who produce soybean and/or corn are more likely to use insurance. On the other hand, the higher the farmers’ propensity to take risk, the lower the likelihood of using insurance [54].

### 2.7. Summary and Prospect

Through elucidating the relevant literature, it is found that AI has a significant positive impact on AO, and it also affects AP behavior, while promoting the income of farmers. The conclusions of most literature show that AI is of great significance to AEG. However, most of the empirical articles study the impact of AI on AEG on a macro level, and few use data at the province level. There is a lack of understanding regarding the pre-disaster and post-disaster mechanism of AI. Furthermore, little of the literature includes three different subjects, which are the government, insurance companies, and farmers as whole. Therefore, on the basis of previous literature and the background of the current situation, this paper analyzes the role of AI on AEG from multiple perspectives in the case of Zhejiang Province, China, and puts forward some suggestions to the three different subjects.

## 3. The Theoretical Analysis on the Mechanism of AI Affecting AEG

Based on theories of welfare economics, this paper contains a theoretical analysis on the mechanism of AI affecting AEG before and after disasters.

### 3.1. Welfare Effect of Agricultural Insurance Based on the Theory of Welfare Economics

Welfare economics is an economic theory to study social and economic welfare founded by British economists Hobbes and Pigou in the 1920s. The main content of welfare economics is “the more equal the distribution, the greater the social welfare”, which advocates the equalization of income. Pigou (1924) proposed that the greater the total national income, the greater the social and economic welfare is, and economic welfare depends on the amount and distribution of national income to a certain extent. Therefore, in order to increase economic welfare, the total national production must be increased, and the unequal distribution of national income must be eliminated.

The welfare effect of AI mainly refers to enhancing social welfare by increasing national income and promoting equal distribution. According to the theory of welfare economics, on the one hand, AI guarantees AO, which inevitably increases the total income of farmers. On the other hand, AI can alleviate the ineffective resource allocation of farmers caused by natural disasters, promote the resources to be distributed rationally, attract more production factors to be invested in agriculture, increase the fairness of resource distribution, and further promote the equalization of farmers’ income. AI stabilizes the development of the AE, which guarantees the overall stable development of the national economy, and ultimately improves the overall national welfare level.

**Figure 1:**
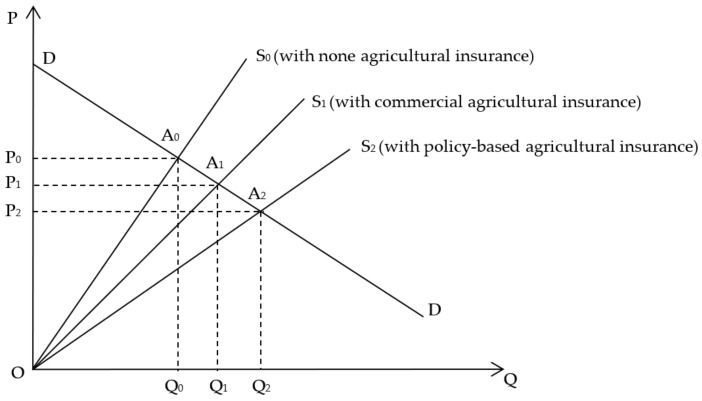
Welfare effect of agricultural insurance.

Figure 1 shows the welfare effect of AI. The vertical axis is the price of agricultural products. The horizontal axis is the quantity of agricultural products. The DD curve is the demand curve of agricultural product, which remains unchanged. The S_0_, S_1_, S_2_ curve is the supply curve of agricultural products when farmers purchase no AI, commercial AI, and PAI, respectively. Figure 1 shows three cases with S_0_, S_1_, and S_2_.

Case 0 is S_0_; farmers do not purchase AI, and the risks of production and operation are borne by themselves. P_0_ is the market balance price of agricultural products with no effect of AI. P_0_ is relatively high. Q_0_ is the balance output of agricultural products. A_0_ is the balance intersection of supply and demand curves. In Case 0, the consumer surplus is P_0_DA_0_, the producer surplus is P_0_OA_0_, and the total social welfare, named social surplus, is ODA_0_.

Case 1 is S_1_; farmers purchase commercial AI, and the supply curve shifts to the right S_1_. A1 is the balance intersection of supply and demand curves. Since AI diversifies the risks of production and operation, so farmers’ risk costs are reduced. The balance price of agricultural products drops to P_1_, and the balance output increases to Q_1_. The total social welfare, named social surplus, expands to ODA_1._ In addition, OA_0_A_1_ is the net increase, which is the social value of introducing unsubsidized commercial AI. There are limitations, as it depends on the farmers’ willingness to use commercial AI. In this case, the premiums of commercial AI are relatively high without government intervention. Some farmers do not take out commercial AI due to the constraints of outcome. This leads to insufficient insurance demand and the cost of insurance premiums is higher, insurance companies are prone to fall into a vicious circle unless the government intervenes.

Case 2 is S_2_; farmers purchase PAI which is subsidized by government, the supply curve continues to shift to the right. A_2_ is the balance intersection of supply and demand curves. The balance price of agricultural products drops to P_2_, and the balance output increases to Q_2_. The total social welfare, named social surplus, expands to ODA_2_ as OA_1_A_2_ is the net increase. PAI increases social welfare from both supply and demand. From the perspective of producers, PAI realizes income redistribution through fiscal transfer payments, which makes the distribution of national income more even and increases social welfare. On the other hand, consumers can purchase agricultural products at lower prices, which increases social welfare once again. At the same time, PAI can also maintain the order of the AI market and keep premium prices at a normal level.

### 3.2. The Mechanism of Agricultural Insurance Affecting Agricultural Economic Growth

**Figure 2:**
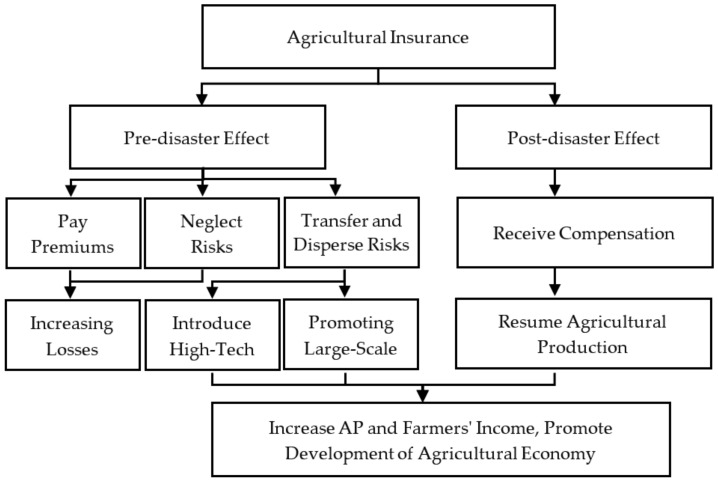
The mechanism of agricultural insurance affecting agricultural economic growth.

AI has both positive and negative impacts on the development of AE. According to the time it is of effect, there are two mechanisms, the pre-disaster effect and post-disaster effect, which are shown in Figure 2.

#### 3.2.1. The Pre-Disaster Effect of Agricultural Insurance

The pre-disaster effect has both positive and negative effects.

The positive effect is that AI can effectively transfer and disperse agricultural risks and stabilize the production expectations of farmers. Farmers who have AI do not worry about risk; therefore, they positively improve AP and introduce high-tech production technologies. The promoting of large-scale mechanization and modernization to increase AP and farmers’ income eventually leads the high-quality development of the AE.

The negative effect is that farmers have to pay premiums for insurance; this expenditure is negative on farmers’ income. Meanwhile, when the risks are guaranteed to a certain extent by AI, farmers are prone to neglect the prevention of agricultural risks, resulting in moral hazards, increasing the possibility and severity of production accidents, and ultimately increasing losses.

#### 3.2.2. The Post-Disaster Effect of Agricultural Insurance

The post-disaster effect is mainly positive. AI has a welfare effect, which can enable insured farmers to receive a certain amount of compensation when there are natural disasters. This enables farmers keep their income without losses and resume AP in a short time. AI can stabilize agricultural reproduction, ensure the sustainability of AP, and promote the stable development of the AE.

## 4. The Status Quo of Agricultural Insurance and Agricultural Economic Growth in Zhejiang Province

In recent years, Zhejiang’s agriculture has continued to move towards a modern agricultural development model, accelerating the realization of agricultural specialization, large-scale production, and marketization. The scale, amount, and coverage of AI have increased significantly. Zhejiang’s AI has achieved leapfrog development.

### 4.1. Current Situation of Agricultural Insurance in Zhejiang Province

Zhejiang is located in a coastal area of east China. There is a lot of rain and rivers, and natural disasters occur frequently. Disasters such as typhoons, floods, landslides, mudslides, snow disasters, and freezing temperatures have occurred to varying degrees. Affected by natural disasters, farmers cannot help but suffer huge disaster losses, which seriously affects the development of rural and agricultural economies and the improvement of farmers’ lives. Therefore, the government of Zhejiang Province attaches great importance to improving the AI system, improving the agricultural risk management and control mechanism, and improving the ability of agriculture to resist risks.

#### 4.1.1. Agricultural Insurance Premium Income and Compensation Expenses

**Figure 3:**
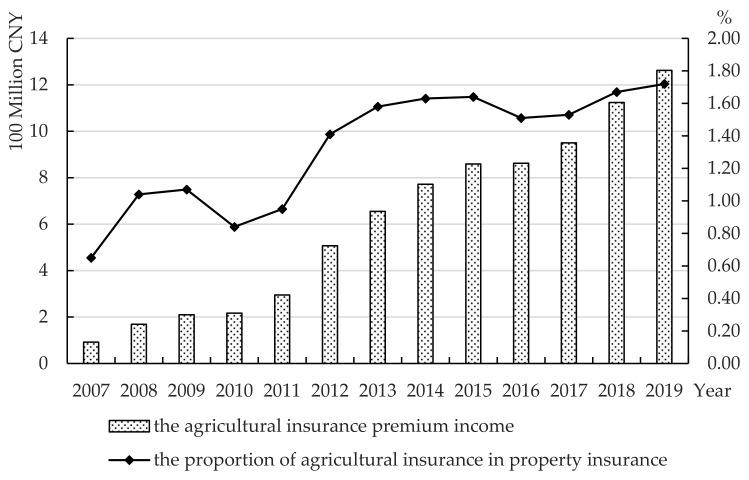
Agricultural insurance premium income and the proportion of agricultural insurance premiums in Zhejiang Province from 2007 to 2019. Data source: China Insurance Yearbook 2020.

Figure 3 is the AIPI and the proportion of AI in property insurance in Zhejiang Province from 2007 to 2019. The trend is increasing, generally. In 2019, AIPI reached 1.262 billion CNY, which is 13 times the premium income in 2007. From 2007 to 2010, the growth rate of premium income tended to be at medium-high speed, while from 2011 to 2015, it increased rapidly. There was a brief decline in 2016, then it resumed the trend of rapid growth from 2017 to 2019. The AI premium revenue scale exceeded 1.1 billion CNY in 2018 and 1.2 billion CNY in 2019. The proportion of AIPI to property insurance premium income in Zhejiang Province fluctuated significantly from 2007 to 2019. However, it shows an upward trend overall, though it is still at a low level at present. In 2019, the proportion accounted for no more than 2%, and it has much room for development.

Table 1 is the AIPI in each city of Zhejiang Province in 2019. There was a large gap and imbalance between these cities. The total AIPI in Hangzhou and Ningbo is close to half of Zhejiang Province’s total premium income, while the development level of AI in Wenzhou, Huzhou, Lishui, Taizhou, and other cities is relatively low, each accounting for about 6% of Zhejiang Province’s total premium income. This shows that there are regional differences in the development level of AI in each city of Zhejiang Province.

**Figure 4:**
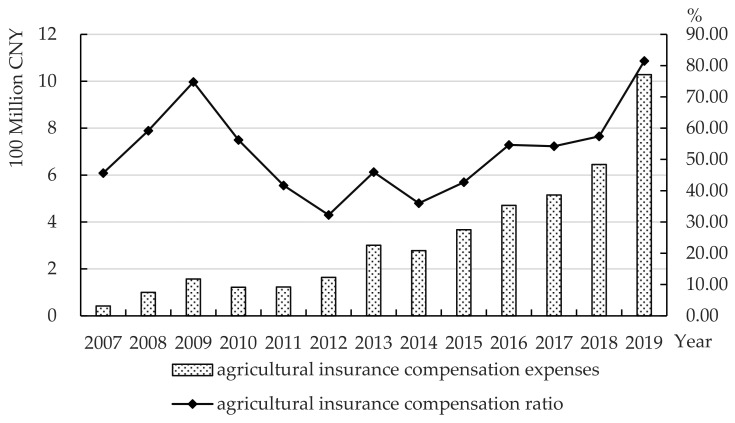
Agricultural insurance compensation expenses and compensation ratio in Zhejiang Province from 2007 to 2019. Data source: China Insurance Yearbook 2020.

Figure 4 shows the AI compensation expenses and compensation ratio in Zhejiang Province from 2007 to 2019. The AI compensation expenditure in Zhejiang Province showed a rapid growth trend, from less than 100 million CNY in 2007 to more than 1 billion CNY in 2019, which reflects the rapid development of AI in Zhejiang Province. The compensation ratio fluctuated greatly. It dropped sharply from 2009 to 2012, and once fell to 32.37% in 2012. From 2014 to 2019, it showed a fluctuating upward trend. At the end of 2019, the AI compensation ratio exceeded 80%.

#### 4.1.2. The Coverage of Agricultural Insurance in Zhejiang Province

In recent years, the coverage of AI in Zhejiang Province has continued to expand. The types of protection have expanded from major agricultural products, such as staple crops and live pigs, to economic varieties, such as tea and fruit. In response to local needs, Zhejiang Province vigorously encourages the development of local types of AI, innovatively develops yield insurance, such as fresh peach, index insurance, such as tea, loquat, bayberry, pear, pecan, and other types of AI. The local types of AI cover all fields of agriculture, forestry, animal husbandry, and fishery, serving the demands of “Multiple products in one county”. Insurance coverage has been continuously improved, and the coverage of more than 10 types of insurance, such as rice and live pigs, has gradually increased. As of 2020, the maximum insurance coverage for rice is 1000 CNY/mu, the coverage for live pigs is 1200 CNY/head, and the coverage for reproductive sows is 1500 CNY/head, which is at a relatively high level in China. Insurance liability continues to expand, basically including major risks, such as natural disasters, pests, weeds, and rodents in Zhejiang Province.

#### 4.1.3. Agricultural Insurance Policies in Zhejiang Province

Zhejiang Province announced the implementation of the “Regulations on Agricultural Insurance” on 15 January 2015. In light of the actual situation of “one village and one product” in rural areas, each city is given full autonomy and encouraged to develop AI with regional characteristics. On 26 October 2018, Zhejiang Province issued the “Opinions on Strengthening Policy-Based Fishery Mutual Insurance Work”, proposing to continuously improve the level of protection for fishery production risks, help fishermen increase their incomes, and protect the healthy development of fisheries. On 26 August 2021, Zhejiang Province issued the “Implementation Opinions on Accelerating the High-quality Development of Agricultural Insurance”, which, formulated in combination with the actual development of AI in Zhejiang Province, proposes to promote the continuous improvement of AI quality and efficiency, the transformation and upgrade from cost insurance to income guarantee, and from a single production link to a complete industrial chain. It is also proposed to allow AI to effectively reducing AP risks fully, ensuring agricultural stability and increasing farmers’ income, and promoting the high-quality development of AI in Zhejiang Province.

### 4.2. Current Situation of Agricultural Economic Development in Zhejiang Province

In recent years, Zhejiang Province has fully implemented the Rural Revitalization Strategy, insisting on giving priority to the development of agriculture and rural areas. In order to accelerate the realization of agricultural modernization, with the important instructions of the Party Central Committee on the work of agriculture, rural areas, and farmers, Zhejiang Province established a rural agricultural big data system, which vigorously promotes smart agriculture development and further liberates the agricultural productive forces, accelerates the transformation of agriculture, and promotes the rapid development of the AE.

#### 4.2.1. The Total Agricultural Output Value of Zhejiang Province

**Figure 5:**
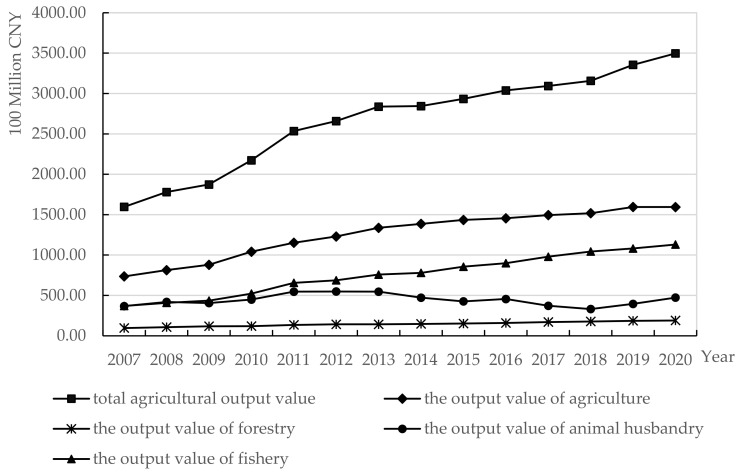
2007–2020 Zhejiang Province’s total agricultural output value and the output value of agricultural, forestry, animal husbandry, and fishery. Data source: Data Zhejiang. http://data.tjj.zj.gov.cn/page/systemmanager/admin/homePage.jsp?orgCode=33 (accessed on 2 April 2022).

Figure 5 is Zhejiang Province’s total AO value and the output value of agricultural, forestry, animal husbandry, and fishery from 2007 to 2020. The trend of total AO value of Zhejiang Province is rising. From 2007 to 2020, the total AO value of Zhejiang Province increased year by year, and the output value of agriculture, forestry, and fishery showed an upward trend. The output value of animal husbandry fluctuated slightly during this period, but it also tended to rise since 2018.

Table 2 is the total AO value and ratio of cities in Zhejiang Province in 2020. The total AO value of Ningbo is the highest at 53.408 billion CNY, accounting for 15.55% of Zhejiang Province’s output value, followed by Taizhou and Hangzhou; the common characteristics of these high-value cities are a high level of agricultural industrialization, large-scale production, and modernization.

#### 4.2.2. Per Capita Disposable Income of Rural Residents in Zhejiang Province

**Figure 6:**
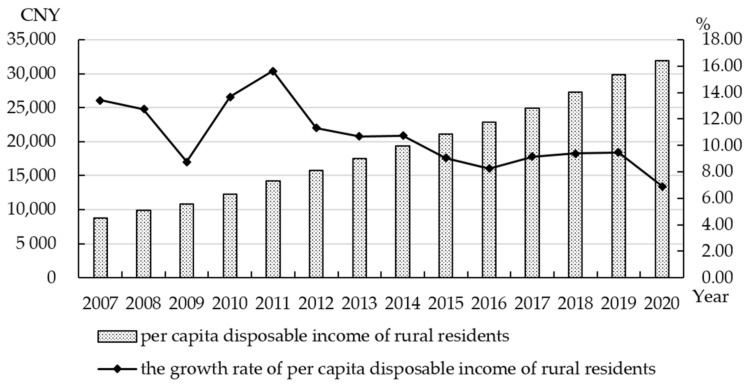
Per capita disposable income and growth rate of rural residents in Zhejiang Province from 2007 to 2020. Data source: Data Zhejiang. http://data.tjj.zj.gov.cn/page/systemmanager/admin/homePage.jsp?orgCode=33 (accessed on 2 April 2022).

The per capita disposable income of rural residents is an important indicator to measure the quality and efficiency of agricultural economic development. Figure 6 shows the per capita disposable income of rural residents in Zhejiang Province continued to increase, from 8805 CNY in 2007 to 31,930 CNY in 2020, which is an increase of 2.6 times. The growth rate of per capita disposable income of rural residents fluctuated greatly from 2007 to 2015, and has remained at 7% to 9% from 2016 to 2020. The income of rural residents increased steadily in general.

## 5. Empirical Analysis of Agricultural Insurance’s Influence on Agricultural Economic Growth

### 5.1. Variable Selection and Data Source

#### 5.1.1. Variable Selection

This paper mainly analyzes the influence of AI on AEG in Zhejiang Province. Therefore, gross output value of agriculture, forestry, animal husbandry, and fishery (GOVA) of 11 cities in Zhejiang Province is selected as the explained variable. The AIPI is selected as the core explanatory variable. The waterlogging area, REC, TPAM, and CSA are selected as control variables.

Explained variable y: GOVA equal to the total amount of agricultural, forestry, animal husbandry, and fishery products which is measured in currency. This variable reflects the total scale of AP in a certain period of time, as it is a measurement of agricultural economic development. It is also the most intuitive and an important indicator to reflect the boosting effect of AI.

Explanatory variable *x*_1_: AIPI refers to the AI expenditures that farmers invest in order to diversify agricultural risks and protect AP and business activities. It is beneficial to protect the stability of AO. This paper mainly studies the impact of AI on AEG. Therefore, AIPI is selected as the core explanatory variable of this paper.

Control variable *x*_2_: AWR refers to the area of farmland where the waterlogging-prone cultivated land is exempted from waterlogging due to the construction of waterlogging control projects or the installation of waterlogging machinery and other water conservancy facilities. The waterlogging removal standard reaches waterlogging excess once in three years. Only by continuously improving the construction of water conservancy and flood control facilities can we enhance the ability to resist disasters and promote the development of modern agriculture. Therefore, AWR is selected as a control variable in this paper.

Control variable *x*_3_: REC refers to the total electricity consumption of rural production and living, which is calculated by deducting the electricity consumption of state-owned industry, transportation, infrastructure, and other state-owned entities in the countryside. Agricultural electrification promotes the development of agricultural modernization, and REC can be used as a control variable to measure the impact of agricultural electrification on AO.

Control variable *x*_4_: TPAM refers to the total power of various power machinery mainly used in agriculture, forestry, animal husbandry, and fishery. Mechanization promotes more professional AP, which can improve the efficiency of agricultural activities, and promote agricultural economic development. TPAM can be used as a control variable to measure the impact of the scale of agricultural mechanization on AO.

Control variable *x*_5_: CSA refers to the area actually sown or transplanted with crops at the end of a certain production season. China is a large agricultural country, and the crop yield depends to a large extent on the sown area of land. Therefore, this paper chooses the CSA as a control variable to measure the impact of land input on AO.

#### 5.1.2. Data Source

The data for the GOVA, the explanatory variables in this paper, the control variables of AWR, REC, TPAM, and CSA are obtained from the statistical yearbook of the Bureau of Statistics of Zhejiang Province, and the missing data for some years are obtained from the statistical yearbook of each city of Zhejiang Province. The data for the explanatory variable, AIPI, are obtained from the “China Insurance Yearbook” in Zhejiang Province. Since the latest edition of the “China Insurance Yearbook”, which contains the AIPI data of cities in Zhejiang Province in 2020, has not been released, and detailed data on AIPI in each city cannot be obtained, the data for the variables used in this paper are the annual data of 11 cities in Zhejiang Province from 2007 to 2019.

### 5.2. Proposition Hypothesis and Model Construction

#### 5.2.1. Proposition Hypothesis

This paper makes the following proposition hypotheses:

**Hypothesis** **1.** *When other conditions remain unchanged, AIPI has a positive impact on GOVA.*

**Hypothesis** **2.** *When other conditions remain unchanged, AWR has a positive impact on GOVA.*

**Hypothesis** **3.** *When other conditions remain unchanged, REC has a positive impact on GOVA.*

**Hypothesis** **4.** *When other conditions remain unchanged, TPAM has a positive impact on GOVA.*

**Hypothesis** **5.** *When other conditions remain unchanged, CSA has a positive impact on GOVA.*

#### 5.2.2. Model Construction

The Cobb–Douglas production function (C–D production function) is an economic model mainly used to measure the impact of input such as labor, technical, and capital on output in the production process. The model is set as follows:

(1) $$Y=AL^{\alpha }K^{\beta }$$

In formula (1), *Y* represents the total output value, *A* represents the comprehensive technical level, *L* represents the labor input amount, *K* represents the capital input amount, and *α* and *β* represent the output elasticity coefficient of labor and capital, respectively. In this paper, referring to the C–D production function model, combined with other factors affecting AEG that need to be analyzed. GOVA represents the total AO level. The AIPI represents the production management method of farmers. The CSA measures the land input. The TPAM and REC represent the technical input, and measure the level of agricultural mechanization and agricultural electrification, respectively. The waterlogging area represents the level of water conservancy construction.

The data used in the empirical analysis are panel data. According to the selected relevant variables, combined with the characteristics of the data panel, we establish a panel data regression model. We test for heteroskedasticity: BP test *p*-value = 0.0387 and White’s test *p*-value = 0.0000. There is heteroscedasticity in the data. In order to eliminate the influence of heteroscedasticity, the natural logarithm of the relevant data is taken. The model used is Formula (2):

(2) lnyit=β0+β1lnx1it+β2lnx2it+β3lnx3it+β4lnx4it+β5lnx5it+vit

In Formula (2), *β*_0_ is a constant term. *y* represents the explanatory variable, total output value of agriculture, forestry, animal husbandry, and fishery. *x*_1_ represents the explanatory variable AIPI. *x*_2_, *x*_3_, *x*_4_, and *x*_5_ represent the control variables AWR, REC, TPAM, CSA. *β*_1_, *β*_2_, *β*_3_, *β*_4_, *β*_5_ are the elastic coefficients between the explained variables, explanatory variables, and each control variable. *v_it_* is the random interference term. *y_it_* represents GOVA in the ith city in the t year. x1it represents the AIPI in the ith city in the t year, and so on. The definition of each variable is shown in Table 3.

### 5.3. Empirical Results

#### 5.3.1. Variables

The measurement software used in this paper is Stata/SE V16 (Perpetual Academic License Single user, Beijing Uone Info&Tech Co., Ltd., Beijing, China). The descriptive statistics of variables are shown in Table 4.

#### 5.3.2. Correlation Analysis of Variables

Firstly, we analyzed the correlation of variables before taking out the regression analysis. The results of the correlation coefficient are shown in Table 5. The correlation coefficient of GOVA with AIPI (r = 0.593, *p* < 0.01), with AWR (r = 0.572, *p* < 0.01), with REC (r = 0.777, *p* < 0.01), with TPAM (r = 0.697, *p* < 0.01), and with CSA (r = 0.272, *p* < 0.01). The variables are all significantly positively correlated, thus we can proceed to the subsequent regression analysis.

#### 5.3.3. Multicollinearity Test for Variables

In the correlation analysis of variables, the correlation coefficient between the REC and the TPAM is 0.801 and statistically significant. Therefore, we carry out a multicollinearity test for variables before the regression analysis. The VIF (variance expansion coefficient) test is carried out on the variables. The multicollinearity test results are shown in Table 6. The VIF value of AIPI is 1.54, the VIF value of AWR is 3.794, the VIF value of REC is 6.486, the VIF value of TPAM is 2.582, and the VIF value of CSA is 1.782. The VIF value of each variable is less than 10, indicating that the model has no multicollinearity, as it is well-constructed, so we can proceed to the empirical analysis.

#### 5.3.4. Regression Model Selection

Considering the individual effect and time effect, in addition to the mixed effects regression (1), the fixed effects model (2) and a random effects model (3) should be used for the panel data model regression estimator. The panel estimator results of the three models are shown in Table 7.

The F-test was used to test the individual effect and the time effect, respectively, and the *p* values are both 0.000 and less than 0.05, which indicates that both the fixed effect and the random effect are statistically significant, and both the fixed effect model (2) and the random effect model (3) are better than the mixed OLS model (1). The mixed OLS model (1) can be rejected based on the test results.

Then we used the Hausman test to determine whether to choose a fixed effects model (2) or a random effects model (3). The Hausman test was performed on the fixed effect model and the random effects model, and the *p* value was 0.207, which was greater than 0.05. Therefore, the null hypothesis was accepted, and the random effects model (3) was selected for empirical analysis.

#### 5.3.5. Empirical Result

Table 7 shows that, in the random effects model (3), the influence coefficient of lnx1 on lny is 0.166, which indicates that the AIPI has a significant positive impact on the growth of the GOVA. This means, when other conditions remain unchanged, the 1% increase in AIPI increases the GOVA by 0.166%, accordingly.

According to the empirical results of the random effects model (3), the control variables of REC, TPAM, and CSA are statistically significant for the growth of the GOVA. The elasticity coefficient of REC is 0.325, indicating that, when other conditions remain unchanged, the 1% increase in REC increases the growth of the GOVA by 0.325%. The elastic coefficient of the TPAM is 0.287, which means that, when other conditions remain unchanged, the 1% increase in TPAM can promote the growth of the GOVA to increase by 0.287%. The elasticity coefficient of CSA is −0.281, which means that CSA negatively affects the GOVA. That is, when other conditions remain unchanged, every 1% increase in CSA reduces the GOVA by 0.281%.

The empirical results of the random effects model (3) in Table 7 show that the development of AI in Zhejiang Province from 2007 to 2019 can promotes the growth of AO and has a certain boosting effect on AEG. To a certain extent, AI can diversify agricultural risks and play a role in ensuring AP, but compared with other variables, its impact is smaller. It should expand the coverage of AI and adjust the structure of AI according to the rural revitalization strategy. In addition, REC and TPAM have a significant promotion effect on the growth of AO, which shows that popularizing agricultural electrification and exerting the large-scale effect of machinery can promote AEG in Zhejiang Province.

#### 5.3.6. Lagged Effect Test

According to the random effects model (3), the data of one lag period are used for lagged effect analysis. The lagged effect test results are shown in Table 8. The parameters show that L.*lnx*_1_ has a significant impact on *lny*, with an impact coefficient of 0.135, indicating that the AIPI of one lag period has a significant positive impact on the growth of the GOVA. This means that there is a lagged effect, and the past AIPI has an impact on the current GOVA.

## 6. Conclusions and Recommendations

### 6.1. Conclusions

Through the analysis of the status quo, theoretical mechanisms, and empirical analysis, this paper draws the following conclusions:

First, there are regional differences in the development of AI in Zhejiang Province. This finding is in line with the policy effect being heterogeneous in different regions [22]. In recent years, the level of AI in Zhejiang Province has continuously improved, the premium income increased continuously, and the coverage of AI expanded continuously. However, the proportion of AI premiums in the total property insurance premiums is relatively low. AI remains at a low level.

Second, the AE in Zhejiang Province has developed steadily. Under the guidance of the rural revitalization strategy, the AE of Zhejiang Province has developed steadily in recent years, and the GOVA and the per capita disposable income of rural areas have shown an upward trend. The level of agricultural industrialization, large-scale production, and modernization is high.

Third, AI mainly affects AO through pre-disaster effects and post-disaster effects, thereby affecting AEG. The pre-disaster effect has both positive and negative effects, and the post-disaster effect is mainly positive. The positive effect can promote AP, farmers’ income, and contribute to the high-quality development of the AE. This is in line with [23]. The negative effect is that farmers need to pay premiums for insurance. At the same time, when the risks are guaranteed to a certain extent by AI, farmers are prone to neglect the prevention of agricultural risks, resulting in moral hazards and ultimately increases losses.

Fourth, the development of AI can promote the growth of AO, and has a certain boosting effect on the growth of AE. This is in line with [1]. However, compared with other variables, its impact is smaller. It is not perfect, and the depth of protection still should be expanded. At the same time, there is a lagged effect; that is, the past AIPI has an impact on the current GOVA. Among the control variables, REC and the TPAM can promote the growth of AO; that is, popularizing agricultural electrification and exerting the scale effect of machinery can promote AEG in Zhejiang Province. Therefore, Zhejiang Province should continuously improve the AI system, vigorously promote high-tech agriculture, and adopt the mechanized production model, to promote the development of the AE.

### 6.2. Policy Recommendations

Following theoretical analysis and empirical analysis, this paper believes that the improvement of the AI level can promote the growth of AO, increase the income of farmers, and effectively promote the growth of the AE. Therefore, AI should be vigorously developed to expand its depth and coverage. At the same time, the popularization of agricultural electrification should be accelerated and the level of agricultural mechanization should be improved to promote AEG.

Since 2006, Zhejiang Province has carried out PAI pilot projects and pioneered the “co-insurance system” model. After years of practice, remarkable results have been achieved. However, there are still gaps in the level of AI between regions, and the development level of AI in some cities is relatively low. Farmers lack awareness of risk management and AI-related systems, and the insurance structure is still not perfect, and the depth of protection still needs to be better understood to increase the farmers’ confidence in insurance companies. To solve the above shortages, we propose the following recommendations.

#### 6.2.1. Recommendations for Government

First, combine government subsidies with regional characteristics, optimizing the structure of financial subsidies. According to local conditions, differentiated policy subsidies should be made according to regional characteristics [55]. The unbalanced development of various regions lead to different levels of agricultural development. Gradients should be divided according to certain standards according to the specific conditions of different regions. The government should raise the subsidy standard for farmers to use agricultural equipment [11,39,50]. Different gradients correspond to different standards of premium subsidies, and the subsidy standards can also be refined to different types of insurance [18,22]. At the same time, actively explore diversified subsidy methods other than premium subsidies, expand the coverage of AI, and promote the further development of AI, thereby increasing AO and promoting AEG.

Second, refine the relevant systems of AI and improve the level of AI protection. Zhejiang Province should introduce corresponding local measures for AI according to its own characteristics, clarify the operation mode of AI, the main subject, and the rights and obligations of each main subject, and constantly standardize and improve the relevant system of AI. The underwriting, claim settlement, and risk dispersion of AI are all operated normally under the legal framework [56], thereby further improving the level of AI protection.

Third, strengthen the publicity of AI and raise farmers’ confidence in the insurance company to participate in insurance [2]. Strengthen policy publicity, organize personnel to go to each village, and make full use of broadcasts, leaflets, banners, and slogans to vigorously publicize AI policies. Meanwhile, carry out policy interpretation, guidance on claims, and mobilize farmers to voluntarily apply for insurance. Pay attention to the importance of the peer effect on the behavior of farmers purchasing policy-based planting agricultural insurance [46].

#### 6.2.2. Recommendations for Insurance Companies

Insurance companies need to focus on developing innovative AI products with broad coverage [4], while technology empowers AI.

Insurance companies should focus on developing insurance products with broad coverage and strong inclusiveness to meet farmers’ basic security demands for AI within the scope of farmers’ ability to pay [6,17]. Prices and security levels are adapted to the needs of farmers with different production scales and income levels [10,57]. A new AI design could be considered specifically for different farmer groups [31].

Insurance companies need to make good use of information technology to innovate AI products and use information technology to scientifically determine rates [58], prevent moral hazard, and manage and control underwriting risks; expand sales channels, give full play to the protection function of AI products, and help rural revitalization strategies.

#### 6.2.3. Recommendations for Farmers

Farmers need to strengthen their awareness of agricultural risk [59], and speed up their adaptation to large-scale and mechanized agriculture.

Accelerate the adaptation to the agricultural clustering, large-scale, and industrialized development model. It can concentrate rural surplus labor, use land on a large scale, and cluster production, enhance the integrity of regional production, and improve the ability to resist risks. At the same time, speed up the pace of agricultural electrification, introduce advanced production technology, adopt new agricultural machinery, improve the labor productivity of farmers, promote AP, and increase the income of farmers, thereby promoting AEG.

### 6.3. Research Deficiencies

Although some innovations have been made by combing the existing literature, there are still deficiencies. For example, in the selection of control variables, although the study pursues the comprehensiveness of the topic, there may still be other factors that have a certain impact on AEG that are missed. In the control variables selection, disaster resistance of crop structure, frequency of natural disasters, etc., may be considered, which have regional heterogeneity and have different effects on AEG. The study proposes that future studies should explore the regional heterogeneity or threshold effects of AI on AEG.

## Figures and Tables

**Table 1:** Agricultural insurance premium income in each city of Zhejiang Province in 2019.

City	AIPI (Million CNY)	Proportion (%)
Hangzhou	290.73	23.03
Ningbo	266.26	21.09
Quzhou	121	9.58
Jiaxing	104.2	8.25
Jinhua	95.74	7.58
Huzhou	82.64	6.55
Shaoxing	79.76	6.32
Taizhou	78.69	6.23
Wenzhou	76.52	6.06
Lishui	53.19	4.21
Zhoushan	13.71	1.09

Data source: China Insurance Yearbook 2020.

**Table 2:** The total agricultural output value of cities in Zhejiang Province in 2020.

City	Total AO (100 Million CNY)	Proportion (%)
Ningbo	534.08	15.55
Taizhou	520.03	15.14
Hangzhou	500.65	14.58
Shaoxing	331.39	9.65
Zhoushan	280.88	8.18
Wenzhou	254.06	7.40
Jinhua	253.01	7.37
Huzhou	238.94	6.96
Jiaxing	211.46	6.16
Lishui	161.78	4.71
Quzhou	148.33	4.32

Data source: 2021 Statistical Yearbook of Cities in Zhejiang Province.

**Table 3:** Definition of variables.

Variable	Abbreviations	Definition
*y*	GOVA	Gross output value of agriculture, forestry, animal husbandry, and fishery, which is equal to the total amount of agricultural, forestry, animal husbandry, and fishery products measured in currency.
*x*_1_	AIPI	Agricultural insurance premium income, which is the agricultural insurance expenditures that farmers invest in order to diversify agricultural risks and protect agricultural production and business activities.
*x*_2_	AWR	The area of waterlogging removal, which refers to the area of farmland where the waterlogging-prone cultivated land is exempted from waterlogging due to the construction of waterlogging control projects or the installation of waterlogging machinery and other water conservancy facilities.
*x*_3_	REC	Rural electricity consumption, which refers to the total electricity consumption of rural production and living, deducting the electricity consumption of state-owned industry, transportation, infrastructure, and other state-owned entities in the countryside.
*x*_4_	TPAM	Total power of agricultural machinery, which refers to the total power of various power machinery mainly used in agriculture, forestry, animal husbandry, and fishery.
*x*_5_	CSA	Crop-sown area, which refers to the area actually sown or transplanted with crops at the end of a certain production season.

**Table 4:** Descriptive statistics of variables.

Variable	Observations	Mean	Std. Dev.	Minimum	Maximum
*ny*	143	5.363	0.459	4.348	6.229
*lnx*_1_	143	3.465	1.202	0.077	5.672
*lnx*_2_	143	3.337	1.203	0.920	4.897
*lnx*_3_	143	3.862	1.166	1.356	5.459
*lnx*_4_	143	5.282	0.364	4.539	6.143
*lnx*_5_	143	5.276	0.764	2.599	6.000

Data source: 2008–2020 Statistical Yearbook of Cities in Zhejiang Province. China Insurance Yearbook 2020. Data Zhejiang. http://data.tjj.zj.gov.cn/page/systemmanager/admin/homePage.jsp?orgCode=33 (accessed on 2 April 2022).

**Table 5:** Correlation analysis of variables.

Variable	*lny*	*lnx*_1_	*lnx*_2_	*lnx*_3_	*lnx*_4_	*lnx*_5_
*lny*	1.000					
*lnx*_1_	0.593 ***	1.000				
*lnx*_2_	0.572 ***	0.359 ***	1.000			
*lnx*_3_	0.777 ***	0.448 ***	0.801 ***	1.000		
*lnx*_4_	0.697 ***	0.241 ***	0.369 ***	0.700 ***	1.000	
*lnx*_5_	0.272 ***	0.532 ***	0.528 ***	0.532 ***	0.380 ***	1.000

*** means *p* < 0.01. Standard errors in (). Data source: 2008–2020 Statistical Yearbook of Cities in Zhejiang Province. China Insurance Yearbook 2020. Data Zhejiang. http://data.tjj.zj.gov.cn/page/systemmanager/admin/homePage.jsp?orgCode=33 (accessed on 2 April 2022).

**Table 6:** Multicollinearity test results.

Variable	VIF	1/VIF
*lnx*_3_	6.486	0.154
*lnx*_2_	3.794	0.264
*lnx*_4_	2.582	0.387
*lnx*_5_	1.782	0.561
*lnx*_1_	1.540	0.649
Mean VIF	3.237	

Data source: 2008–2020 Statistical Yearbook of Cities in Zhejiang Province. China Insurance Yearbook 2020. Data Zhejiang. http://data.tjj.zj.gov.cn/page/systemmanager/admin/homePage.jsp?orgCode=33 (accessed on 2 April 2022).

**Table 7:** Regression results of panel data model.

Variable	(1) Mixed Effects Parameters	(2) Fixed Effects Parameters	(3) Random Effects Parameters
*lnx*_1_	0.196 ***	0.156 ***	0.166 ***
	(0.016)	(0.019)	(0.015)
*lnx*_2_	0.090 ***	−0.218	−0.050
	(0.024)	(0.130)	(0.066)
*lnx*_3_	0.105 **	0.411 ***	0.325 ***
	(0.033)	(0.098)	(0.065)
*lnx*_4_	0.594 ***	0.296 **	0.287 ***
	(0.067)	(0.088)	(0.079)
*lnx*_5_	−0.268 ***	−0.343 **	−0.281 ***
	(0.026)	(0.110)	(0.065)
*cons*	2.257 ***	4.212 ***	3.666 ***
	(0.303)	(0.864)	(0.428)
*Observations*	143	143	143
*R-squared*	0.853	0.814	0.810
*Prob (F-statistic)*		0.000	0.000
*Prob (Hausman)*		0.207	

*** *p* < 0.01, ** *p* < 0.05. Standard errors in (). Data source: 2008–2020 Statistical Yearbook of Cities in Zhejiang Province. China Insurance Yearbook 2020. Data Zhejiang. http://data.tjj.zj.gov.cn/page/systemmanager/admin/homePage.jsp?orgCode=33 (accessed on 2 April 2022).

**Table 8:** Lagged effect test results.

Variable	Random Effects Parameters	Lag 1 Period Parameter
*lnx*_1_	0.166 ***	
	(0.0147)	
*lnx*_2_	−0.0504	−0.0901
	(0.0660)	(0.0696)
*lnx*_3_	0.325 ***	0.375 ***
	(0.0652)	(0.0689)
*lnx*_4_	0.287 ***	0.218 *
	(0.0791)	(0.0993)
*lnx*_5_	−0.281 ***	−0.249 ***
	(0.0651)	(0.0702)
L.*lnx*_1_		0.135 ***
		(0.0164)
*_cons*	3.666 ***	3.947 ***
	(0.428)	(0.510)
*Observations*	143	132

*** *p* < 0.01, * *p* < 0.1. Standard errors in (). Data source: 2008–2020 Statistical Yearbook of Cities in Zhejiang Province. China Insurance Yearbook 2020. Data Zhejiang. http://data.tjj.zj.gov.cn/page/systemmanager/admin/homePage.jsp?orgCode=33 (accessed on 2 April 2022).

## Abbreviations

**Abbreviations**	**Key Term**
AI	(the) agricultural insurance
AEG	agricultural economic growth
AP	agricultural production
AO	agricultural output
AE	agricultural economy
AIPI	agricultural insurance premium income
REC	rural electricity consumption
TPAM	total power of agricultural machinery
CSA	crop-sown area
AGTFP	agricultural green total factor productivity
PAI	policy-based agricultural insurance
GOVA	gross output value of agriculture, forestry, animal husbandry, and fishery
AWR	(the) area of waterlogging removal
